# Employments for Women

**Published:** 1892-10-08

**Authors:** 


### Employments for Women.

*(Continued from page 260, Vol. XII.)*

IV.—LECTURING FOR THE COUNTY COUNCILS.

The National Health Society only supplies lecturers, so far as we see, upon the subjects with which it is naturally concerned—hygiene, nursing, and cookery. But the County Councils send out into the country teachers of other subjects also—dairy work, laundry work, domestic sewing, dress cutting, &c., and these can be learned, as also can the first-named subjects, at the Liverpool Technical College for Women (Hon. Sec., Miss Fanny Calder). Here cookery in all its branches is taught, special attention being given to artisan cookery. To gain what is called the artisan diploma the student must pass through a course of cleaning and scullery work, plain household cookery, demonstration and practice lessons in artisan cookery, and, finally, a course of practice in giving demonstration and practice lessons. The fee for this training is seven guineas. The course of "household sewing with home dress-cutting" includes six lessons on dress-cutting, and six to ten on sewing and making-up of dresses, besides twelve lessons on mending, patching, darning, and plain sewing, and six on drawn-thread and fancy stitches, the fee for the course being £4 4s. We may mention that this course takes but a comparatively short time, classes being held every day except Saturdays. The fee for elementary laundry work is also four guineas. Training for "Health in the Home" has just been organized, and arrangements are also made for ambulance classes.

We need hardly say that women wishing to qualify themselves for these lectureships should take up their subjects *con amore*, and do their best to obtain a firm grip of them. There is all the difference in the world between a teacher who gets her lesson by heart, and gives it out in regular routine, now here, now there, as her services are required, and one who throws all her energies into it, puzzling out all she does not fully understand, making experiments for herself, and finally, when her turn to teach comes, showing by her every movement, every glance, that she is thoroughly interested herself, and expects her hearers to be the same. The former may pass all her examinations, and perhaps get plenty of work too; but who can doubt that the result of her work (and this is not a mere matter of earning money, but of doing real good) will be as nothing compared with that accomplished by the latter? We have watched an ambulance class, and have been much struck by the wonderfully different way in which it was taken up by the various members of it—none of them, it may be mentioned, having any idea of utilising their knowledge except when occasion should offer in their own homes. While some only thought of how much they must "get up" in order to pass their examination with decent credit, others were simply engrossed in the subjects—met together to talk over points on which they were doubtful, hunted up the meaning of any unknown word, spent whole afternoons in the public library perusing anatomical works, and, finally, experienced a momentary feeling of disappointment upon finding that the examination papers were to them, after their many weeks' work, mere child's play. These are the sort of women we want for our lectureships; these are the women who will rouse enthusiasm in the poor cottager's heart, and inspire her with a longing to prove to her husband—what he has never believed, often as he may have heard it sung—that "there's no place like home."

But one word more of warning. It does not by any means follow that if we know a subject well, we shall be sure to teach it well. For this we must have imagination as well as intellect; and no one can be a really good teacher who is quite devoid of imagination. It is a faculty, however, which most of us possess in more or less measure, and which can without doubt be cultivated. We must try to put ourselves in the position of ignorant people, to realise that what seems perfectly simple to us will be complex and difficult to them, to remember how small is their vocabulary (we believe it has been said that about nine hundred words form the ordinary vocabulary of a country labourer), and therefore to use very simple words, or at least to couple together a more difficult word and its equivalent in simpler language.

And even when put into a language which can be "understanded of the people," these lectures must be very carefully arranged, or they will only muddle the brains of the hearers. We have just had a description of a country cooking-class, from an educated and very intelligent woman who was present at it. "The lady herself," says our informant, "was charming both to look at and to talk to, but the whole thing was rather a jumble. About eight things were cooked all at once; and for the people who did not understand cooking (myself being one, and therefore able to sympathise with the poor women), it was impossible to remember which quantities were for which article, and into which saucepan the teacher put the last ounce of material. She also prepared a great deal beforehand, all of which the poor people ought to have seen done, such as whipping up eggs. I am sure they did not believe that the plate of solid-looking white stuff was nothing but the white of one egg, whipped. The lady talked well and plainly; it was the quantity of things, and the confusion of ingredients, that spoilt the class."

